# 3-Oxocyclo­butane­carboxylic acid: hydrogen bonding in a small-ring γ-keto acid

**DOI:** 10.1107/S1600536809003961

**Published:** 2009-02-11

**Authors:** Georgia Efthimiopoulos, Hugh W. Thompson, Roger A. Lalancette

**Affiliations:** a Carl A. Olson Memorial Laboratories, Department of Chemistry, Rutgers University, Newark, NJ 07102, USA

## Abstract

The title ketocarboxylic acid, C_5_H_6_O_3_, is the smallest carboxy­cyclanone to have its crystal structure determined. It adopts a chiral conformation, by rotation of its carboxyl O atoms away from the plane of skeletal symmetry that passes through the carboxyl carbon and both atoms of the ketone carbonyl. The four-membered ring is non-planar, with a shallow fold of 14.3 (1)° along a line connecting the two α-carbons of the ketone group. In the crystal, the molecules are linked by centrosymmetric hydrogen-bond pairing of ordered carboxylic acid groups [O⋯O = 2.6392 (12) Å and O—H⋯O = 175.74 (15)°], yielding two different sets of dimers, related by by a 2_1_ screw axis in *c*, in the cell. A C—H⋯O interaction is also present.

## Related literature

For related structures, see: Barcon *et al.* (1999 ▶); Borthwick (1980 ▶); Harata *et al.* (1977 ▶); Malak *et al.* (2006 ▶); Meiboom & Snyder (1967 ▶); Pigou & Schiesser (1988 ▶). For hydrogen bonding, see: Steiner (1997 ▶).
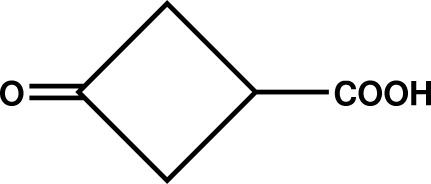


## Experimental

### 

#### Crystal data

C_5_H_6_O_3_
*M* *_r_* = 114.10
Monoclinic, 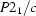

*a* = 8.8858 (19) Å
*b* = 5.3631 (12) Å
*c* = 11.625 (3) Å
β = 106.899 (4)°
*V* = 530.1 (2) Å^3^
*Z* = 4
Cu *K*α radiation
μ = 1.03 mm^−1^
*T* = 100 (2) K
0.48 × 0.20 × 0.15 mm

#### Data collection

Bruker SMART CCD APEXII area-detector diffractometer
Absorption correction: numerical (*SADABS*; Sheldrick, 2008 ▶) *T*_min_ = 0.638, *T*_max_ = 0.861
3854 measured reflections
906 independent reflections
891 reflections with *I* > 2σ(*I*)
*R*_int_ = 0.019

#### Refinement

*R*[*F* ^2^ > 2σ(*F* ^2^)] = 0.029
*wR*(*F* ^2^) = 0.070
*S* = 1.06
906 reflections
77 parameters
H atoms treated by a mixture of independent and constrained refinement
Δρ_max_ = 0.25 e Å^−3^
Δρ_min_ = −0.17 e Å^−3^

### 

Data collection: *APEX2* (Bruker, 2006 ▶); cell refinement: *SAINT* (Bruker, 2005 ▶); data reduction: *SAINT*; program(s) used to solve structure: *SHELXTL* (Sheldrick, 2008 ▶); program(s) used to refine structure: *SHELXTL*; molecular graphics: *SHELXTL*; software used to prepare material for publication: *SHELXTL*.

## Supplementary Material

Crystal structure: contains datablocks I, global. DOI: 10.1107/S1600536809003961/fl2232sup1.cif

Structure factors: contains datablocks I. DOI: 10.1107/S1600536809003961/fl2232Isup2.hkl

Additional supplementary materials:  crystallographic information; 3D view; checkCIF report

## Figures and Tables

**Table 1:** Hydrogen-bond geometry (Å, °)

*D*—H⋯*A*	*D*—H	H⋯*A*	*D*⋯*A*	*D*—H⋯*A*
O3—H3⋯O2^i^	0.885 (18)	1.756 (18)	2.6392 (12)	175.74 (15)
C1—H1⋯O1^ii^	1.00	2.45	3.1003 (15)	122

Symmetry codes: (i) 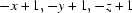; (ii) 
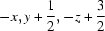
.

**Fig. 1.**
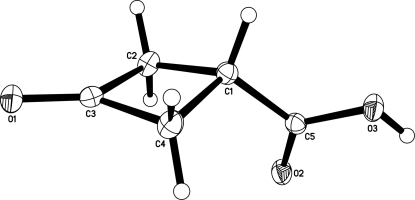
The asymmetric unit for (I). Displacement ellipsoids are drawn at the 40% probability level.

**Fig. 2.**
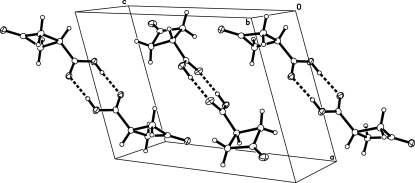
A partial packing diagram, illustrating the centrosymmetric pairing of the asymmetric units, with dimers centered at 1/2,1/2,1/2 and at 1/2,0,0 of the chosen cell. Displacement ellipsoids are drawn at the 40% probability level.

## supplementary crystallographic information

### Comment

Our study of crystalline ketocarboxylic acids explores their five known hydrogen-bonding modes. The most frequently encountered overall is carboxyl pairing, but acid-to-ketone catemers constitute a sizable minority of cases, followed by the three remaining, rarely observed patterns, consisting of acid-to-acid catemers, internal H-bonds and carboxyl-to-ketone dimers. Of significant interest is the behavior of keto acids in the smallest size range, where aggregation influences other than H-bonding are minimized. These may offer insights into the minimum requirements for specific aggregation modes. Among simple C_3_—C_5_ monocarboxyketones, only the (dimeric) crystal structure of pyruvic acid (Harata *et al.*, 1977) has been reported to date, while 3-oxocyclopentanecarboxylic acid (Malak *et al.*, 2006), a catemer, is the sole published example of a C_6_ keto acid.

Fig. 1 depicts the asymmetric unit for the title compound, (I), and its conformation. Although (I) is inherently symmetric, the carboxyl is rotated away from alignment along the central plane of skeletal symmetry, producing a chiral conformation; the O2—C5—C1—C4 torsion angle = 78.70 (14) Å. As is normal in cyclobutanes, the ring is not planar but flexed to ease the eclipsing strain that arises when the torsional angles for substituents on adjacent carbons approach zero. This nonplanarity in (I) may be envisioned as the result of folding the ring along a line connecting C2 to C4; the ketone and carboxyl halves of the ring lie in planes at a mutual dihedral angle of 14.3 (1)°. Because of the presence of the ketone, this dihedral is significantly smaller than those typically seen in cyclobutanes containing only *sp*^3^ carbons, which range from 19.1° in *trans*-pinononic acid (Barcon *et al.*, 1999) to 35° in cyclobutane itself (Meiboom & Snyder, 1967). When any four-sided figure departs from planarity, the internal angles are no longer constrained to an average of 90°, but may approach zero. Because of the shallowness of the fold in (I), the average of all four internal ring angles is 89.63 (18)°, with the carbonyl, where the hybridization strain is greatest, having an angle of 92.88 (9)°. Presumably due to packing interactions, the internal ring angles at C2 and C4 differ slightly, with values of 88.37 (9) and 87.33 (9)°, respectively. The dihedral angle between the carboxyl and ketone planes (O2—O3—C1—C5 *versus* O1—C2—C3—C4) is 69.40 (5)°.

Although disorder-averaging of C—O bond lengths and C—C—O angles is common in dimeric carboxyls, these lengths and angles are not significantly averaged in (I), but conform to values typical of highly ordered cases (Borthwick, 1980).

In contrast to its next-higher ring-homolog (Malak *et al.*, 2006), compound (I) aggregates as standard carboxyl dimers. Fig. 2 illustrates the packing of the chosen cell with the centrosymmetrically hydrogen-bonded pairs of asymmetric units. These appear in two orientations, centered at 1/2,1/2,1/2 and at 1/2,0,0.

Within the 2.6 Å range we standardly survey for C—H···O packing interactions (Steiner, 1997), a single close intermolecular contact was found, involving the ketone (Table 1).

### Experimental

Compound (I) was synthesized in low yield by the method of Pigou & Schiesser (1988); when the final extract failed to crystallize spontaneously on concentration, (I) was isolated by sublimation. Recrystallization from hexane-ether provided material suitable for X-ray, mp 342 K. The solid-state (KBr) infrared spectrum of (I) features widely separated carbonyl peaks for strained ketone and carboxyl dimer, at 1786 & 1696 cm^-1^, respectively. In CHCl_3_ solution, where dimers predominate, the separation is nearly identical, although the peaks are somewhat shifted, at 1797 & 1709 cm^-1^.

### Refinement

All H atoms for (I) were found in electron density difference maps. The carboxyl H was refined positionally with *U*_iso_(H) = 1.5*U*_eq_(O). The methylene and methine Hs were placed in geometrically idealized positions and constrained to ride on their parent C atoms with C—H distances of 0.99 and 1.00 Å, respectively, and *U*_iso_(H) = 1.2*U*_eq_(C).

### Crystal data

**Table:**

C_5_H_6_O_3_	*F*(000) = 240
*M**_r_* = 114.10	*D*_x_ = 1.430 Mg m^−^^3^
Monoclinic, *P*2_1_/*c*	Melting point: 342 K
Hall symbol: -P 2ybc	Cu *K*α radiation, λ = 1.54178 Å
*a* = 8.8858 (19) Å	Cell parameters from 3724 reflections
*b* = 5.3631 (12) Å	θ = 4.0–66.7°
*c* = 11.625 (3) Å	µ = 1.03 mm^−^^1^
β = 106.899 (4)°	*T* = 100 K
*V* = 530.1 (2) Å^3^	Parallelepiped, colourless
*Z* = 4	0.48 × 0.20 × 0.15 mm

### Data collection

**Table:**

Bruker SMART CCD APEXII area-detector diffractometer	906 independent reflections
Radiation source: fine-focus sealed tube	891 reflections with *I* > 2σ(*I*)
graphite	*R*_int_ = 0.019
φ and ω scans	θ_max_ = 66.7°, θ_min_ = 5.2°
Absorption correction: numerical (*SADABS*; Sheldrick, 2008)	*h* = −10→10
*T*_min_ = 0.638, *T*_max_ = 0.861	*k* = −5→6
3854 measured reflections	*l* = −13→13

### Refinement

**Table:**

Refinement on *F*^2^	Secondary atom site location: difference Fourier map
Least-squares matrix: full	Hydrogen site location: inferred from neighbouring sites
*R*[*F*^2^ > 2σ(*F*^2^)] = 0.029	H atoms treated by a mixture of independent and constrained refinement
*wR*(*F*^2^) = 0.070	*w* = 1/[σ^2^(*F*_o_^2^) + (0.0304*P*)^2^ + 0.27*P*] where *P* = (*F*_o_^2^ + 2*F*_c_^2^)/3
*S* = 1.06	(Δ/σ)_max_ < 0.001
906 reflections	Δρ_max_ = 0.25 e Å^−^^3^
77 parameters	Δρ_min_ = −0.17 e Å^−^^3^
0 restraints	Extinction correction: *SHELXTL* (Sheldrick, 2008), Fc^*^=kFc[1+0.001xFc^2^λ^3^/sin(2θ)]^-1/4^
Primary atom site location: structure-invariant direct methods	Extinction coefficient: 0.0140 (13)

### Special details

**Table:**

Experimental. crystal mounted on a Cryoloop using Paratone-N
Geometry. All e.s.d.'s (except the e.s.d. in the dihedral angle between two l.s. planes) are estimated using the full covariance matrix. The cell e.s.d.'s are taken into account individually in the estimation of e.s.d.'s in distances, angles and torsion angles; correlations between e.s.d.'s in cell parameters are only used when they are defined by crystal symmetry. An approximate (isotropic) treatment of cell e.s.d.'s is used for estimating e.s.d.'s involving l.s. planes.
Refinement. Refinement of *F*^2^ against ALL reflections. The weighted *R*-factor *wR* and goodness of fit *S* are based on *F*^2^, conventional *R*-factors *R* are based on *F*, with *F* set to zero for negative *F*^2^. The threshold expression of *F*^2^ > σ(*F*^2^) is used only for calculating *R*-factors(gt) *etc*. and is not relevant to the choice of reflections for refinement. *R*-factors based on *F*^2^ are statistically about twice as large as those based on *F*, and *R*-factors based on ALL data will be even larger.

### Fractional atomic coordinates and isotropic or equivalent isotropic displacement parameters (Å^2^)

**Table:**

	*x*	*y*	*z*	*U*_iso_*/*U*_eq_	
O1	0.11919 (10)	0.09994 (19)	0.84099 (8)	0.0276 (3)	
C1	0.20439 (13)	0.4459 (2)	0.63057 (10)	0.0164 (3)	
H1	0.1194	0.5695	0.5949	0.020*	
O2	0.42464 (10)	0.29447 (16)	0.57144 (8)	0.0226 (3)	
C2	0.13692 (14)	0.1810 (2)	0.63346 (11)	0.0190 (3)	
H2A	0.0251	0.1641	0.5863	0.023*	
H2B	0.2016	0.0472	0.6132	0.023*	
O3	0.35444 (10)	0.69510 (15)	0.53889 (8)	0.0197 (3)	
H3	0.4319 (19)	0.696 (3)	0.5052 (14)	0.030*	
C3	0.16234 (13)	0.2142 (2)	0.76737 (11)	0.0187 (3)	
C4	0.25515 (14)	0.4542 (2)	0.77165 (11)	0.0204 (3)	
H4A	0.3698	0.4359	0.8096	0.024*	
H4B	0.2126	0.5984	0.8053	0.024*	
C5	0.33761 (13)	0.4681 (2)	0.57643 (10)	0.0158 (3)	

### Atomic displacement parameters (Å^2^)

**Table:**

	*U*^11^	*U*^22^	*U*^33^	*U*^12^	*U*^13^	*U*^23^
O1	0.0220 (5)	0.0380 (6)	0.0232 (5)	−0.0043 (4)	0.0075 (4)	0.0098 (4)
C1	0.0165 (6)	0.0169 (6)	0.0165 (6)	0.0006 (4)	0.0056 (4)	0.0008 (5)
O2	0.0221 (5)	0.0194 (5)	0.0304 (5)	0.0050 (4)	0.0140 (4)	0.0079 (4)
C2	0.0194 (6)	0.0187 (7)	0.0206 (6)	−0.0032 (5)	0.0085 (5)	−0.0026 (5)
O3	0.0222 (5)	0.0155 (5)	0.0254 (5)	0.0003 (3)	0.0132 (4)	0.0021 (3)
C3	0.0135 (6)	0.0229 (7)	0.0198 (6)	0.0018 (5)	0.0049 (4)	0.0033 (5)
C4	0.0208 (6)	0.0253 (7)	0.0162 (6)	−0.0041 (5)	0.0071 (5)	−0.0035 (5)
C5	0.0165 (6)	0.0168 (6)	0.0132 (5)	−0.0004 (5)	0.0028 (4)	0.0006 (4)

### Geometric parameters (Å, °)

**Table:**

O1—C3	1.2024 (15)	C2—H2A	0.9900
C1—C5	1.4983 (16)	C2—H2B	0.9900
C1—C2	1.5458 (17)	O3—C5	1.3164 (15)
C1—C4	1.5701 (16)	O3—H3	0.885 (18)
C1—H1	1.0000	C3—C4	1.5217 (17)
O2—C5	1.2225 (15)	C4—H4A	0.9900
C2—C3	1.5171 (17)	C4—H4B	0.9900
			
C5—C1—C2	116.17 (10)	O1—C3—C2	133.43 (12)
C5—C1—C4	114.48 (9)	O1—C3—C4	133.61 (12)
C2—C1—C4	89.94 (9)	C2—C3—C4	92.88 (9)
C5—C1—H1	111.5	C3—C4—C1	87.33 (9)
C2—C1—H1	111.5	C3—C4—H4A	114.1
C4—C1—H1	111.5	C1—C4—H4A	114.1
C3—C2—C1	88.37 (9)	C3—C4—H4B	114.1
C3—C2—H2A	113.9	C1—C4—H4B	114.1
C1—C2—H2A	113.9	H4A—C4—H4B	111.3
C3—C2—H2B	113.9	O2—C5—O3	123.66 (11)
C1—C2—H2B	113.9	O2—C5—C1	123.18 (11)
H2A—C2—H2B	111.1	O3—C5—C1	113.12 (10)
C5—O3—H3	109.3 (10)		
			
C5—C1—C2—C3	126.54 (10)	C5—C1—C4—C3	−128.00 (10)
C4—C1—C2—C3	9.07 (8)	C2—C1—C4—C3	−9.05 (8)
C1—C2—C3—O1	167.67 (14)	C2—C1—C5—O2	−24.15 (16)
C1—C2—C3—C4	−9.38 (9)	C4—C1—C5—O2	78.70 (14)
O1—C3—C4—C1	−167.80 (14)	C2—C1—C5—O3	158.11 (10)
C2—C3—C4—C1	9.24 (9)	C4—C1—C5—O3	−99.04 (12)

### Hydrogen-bond geometry (Å, °)

**Table:**

*D*—H···*A*	*D*—H	H···*A*	*D*···*A*	*D*—H···*A*
O3—H3···O2^i^	0.885 (18)	1.756 (18)	2.6392 (12)	175.74 (15)
C1—H1···O1^ii^	1.00	2.45	3.1003 (15)	122

Symmetry codes: (i) −*x*+1, −*y*+1, −*z*+1; (ii) −*x*, *y*+1/2, −*z*+3/2.
